# Anesthetic management in pregnancy with osteogenesis imperfecta type XI: A comprehensive case report

**DOI:** 10.1016/j.ijscr.2025.110971

**Published:** 2025-02-01

**Authors:** Korakod Punnaniti, Lisa Sangkum, Sivaporn Termpornlert, Rojnarin Komonhirun, Sasima Dusitkasem

**Affiliations:** Department of Anesthesiology, Faculty of Medicine Ramathibodi Hospital, Mahidol University, Bangkok, Thailand

**Keywords:** Bruck syndrome, Caesarean delivery, FKBP10 mutation, General anesthesia, Osteogenesis imperfecta (OI) type XI

## Abstract

**Introduction:**

Osteogenesis imperfecta (OI) type XI or Bruck syndrome is an extremely rare genetic disorder characterized by congenital joint contractures and bone fragility. OI presents unique and considerable challenges in the perioperative and anesthetic management of affected patients.

**Presentation of case:**

A 29-year-old primigravida with OI type XI (100 cm, 26 kg) and severe kyphoscoliosis underwent urgent Caesarean delivery at 32 weeks under general anesthesia (sevoflurane/nitrous oxide). A female infant (1470 g) required resuscitation. Postoperative recovery was uneventful.

**Discussion:**

The rarity of this syndrome, along with the physiological changes associated with pregnancy, creates an unprecedented clinical scenario that demands a thorough and cautious approach to patient care.

**Conclusion:**

Osteogenesis imperfecta (OI) type XI necessitates careful anesthetic management in pregnancy. This case highlights the anesthetic management challenges and the use of a multidisciplinary approach to enhance clinical understanding and improve patient outcomes.

## Highlights

• A rare case of Caesarean delivery in a patient with Osteogenesis Imperfecta type XI
• Severe kyphoscoliosis and restrictive lung disease complicated anesthesia management.
• General anesthesia was chosen due to vertebral malformations precluding neuraxial blocks.
• Multidisciplinary team approach ensured safe delivery and minimized perioperative risks.
• Successful maternal recovery and neonatal outcome despite significant clinical challenges

## 1 Introduction

Osteogenesis imperfecta (OI), commonly known as brittle bone disease, is a genetic disorder primarily affecting the synthesis of type I collagen, leading to easily fractured bones [1]. The condition is heterogeneous, with varying degrees of severity classified into various types [2]. OI Type XI or Bruck syndrome is a rare autosomal-recessive disease caused by an FKBP10 mutation [3]. It is characterized by long bone fractures, laxity of the ligaments, multiple joint contractures (or stiffness), scoliosis and flattened vertebral bodies [4,5]. Due to its moderately severe and progressive nature, FKBP65-related OI most closely resembles OI type III; however, the phenotype does not fit neatly into this category [6]. To date, few studies have discussed the anesthetic care of individuals with OI type XI, particularly in obstetrics. We present this novel case to contribute to the comprehensive perioperative care required for such patients, emphasizing the importance of a multidisciplinary approach to optimize both maternal and fetal outcomes. This case report is reported according to SCARE guideline [7].

## 2 Presentation of case

**Fig. 1:**
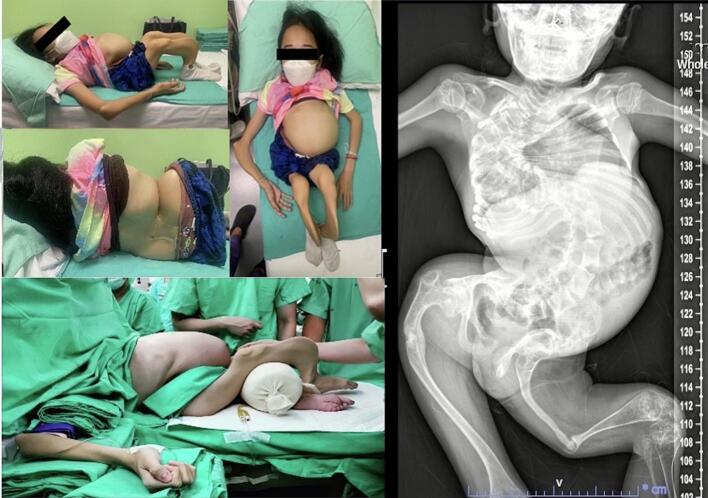
Characteristics of the patient showing multiple joint contractures and deformities.

A 29-year-old woman, primigravida, with OI type XI was referred to our anesthesiology department at 19 weeks' gestation. She was 100 cm tall, weighed 26 kg and had severe disproportionately short stature, grey sclera, a small chest and bowing of her lower limbs (Fig. 1). She had a history of multiple bone fractures after minor trauma, severe kyphoscoliosis and severe restrictive lung disease. She had previously undergone spinal correction and prosthetic removal procedures. Previous anesthetic records revealed uneventful surgery and successful endotracheal tube intubation in one attempt by using a video laryngoscope. Pharmacological treatment included the administration of calcium and vitamin D.

**Fig. 2:**
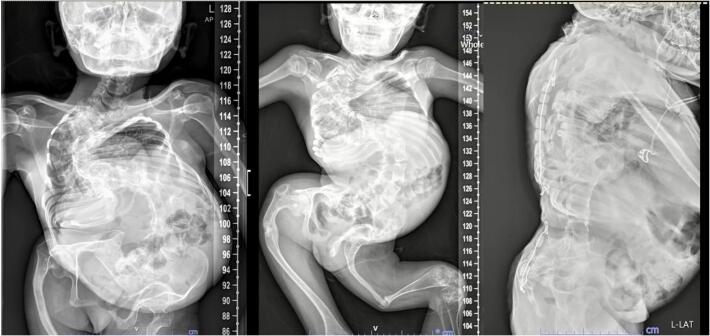
Whole spine X-ray showing diffuse osteopenia and severe scoliosis with right thoracic and left lumbar convexities, causing relatively decreased volume of the right hemithorax and abnormal thoracic cage.

A comprehensive preoperative assessment was planned, and a multidisciplinary team was assembled. The patient's airway assessment was classified as Mallampati class I. The patient's blood pressure was 100/60 mmHg, heart rate was 100 beats/min, respiratory rate was 22 breaths/min. Auscultation of her lungs was unremarkable, and oxygen saturation was 97 % on room air. A previous chest radiograph showed severe kyphoscoliosis of the thoracic and lumbar spine (Fig. 2). Prepregnant pulmonary function tests indicated severe restrictive lung disease. Baseline arterial blood gas analysis showed the following: pH: 7.40, partial pressure of carbon dioxide: 32.7 mmHg, partial pressure of oxygen: 83 mmHg, bicarbonate: 20.3 mEq/l, base excess: −2.5 mEq/l. Echocardiography revealed normal biventricular systolic function, mild mitral regurgitation and low probability of pulmonary hypertension. The results of all routine laboratory tests were normal.

The patient was closely monitored in-hospital, and Caesarean delivery was scheduled for gestational week 34. Nocturnal bilevel positive airway pressure ventilation was provided to ensure the patient's comfort. A Caesarean birth simulation scenario was created and rehearsed step-by-step. Special pillows and pads were created to accommodate the patient's unique anatomy. This preparation process allowed optimization of teamwork, communication, and patient safety, which were integral to the multidisciplinary care from before to after surgery. The simulation was recorded video to be distributed to the remaining team members. Her prenatal course was uneventful, and the growing uterus had only minimal impact on her comfort and respiratory status. Spontaneous onset of labor occurred at 32 weeks' gestation, and she underwent urgent Caesarean delivery.

Handling and positioning of patients with OI require special attention to prevent fractures and other complications. Bone fractures can occur from inadvertent actions such as leaning on the patient, placing heavy instruments on them, or moving them between the stretcher and the operating table. To avoid such injuries, after arrival in the operating room, we asked our patient to move herself onto the operating table. All pressure points are properly cushioned. Effective patient care underscores the need for a multidisciplinary approach, involving close collaboration with orthopedic specialists to strategize safe positioning and careful handling throughout the entire perioperative period. The patient's initial blood pressure was 96/63 mmHg, heart rate was 103 beats/min, respiratory rate was 20/min and blood oxygen saturation was 97 %. Arterial lines were inserted. Rapid-sequence induction was performed using intravenous propofol 2.5 mg kg^−1^, lidocaine 1 mg kg^−1^ and rocuronium 1.2 mg kg^−1^. A 6.5-mm cuffed endotracheal tube was placed using a video laryngoscope. General anesthesia was maintained initially with nitrous oxide, oxygen and sevoflurane. The tidal volume was set at approximately 240–260 ml; respiratory rate: 18 breaths/min, and positive end-expiratory pressure: 5 cm H2O. Intraoperative peak inspiratory pressures ranged from 20 to 25 cm H20. The patient's hemodynamic parameters remained stable, and the operation was uneventful. A female infant weighing 1470 g was delivered, and the Apgar scores were 2, 5 and 9 at 1, 5 and 10 min, respectively. Accordingly, the infant required early resuscitation and was transferred to the neonatal intensive care unit. After umbilical cord clamping, intravenous carbetocin 100 IU, midazolam 0.1 mg kg^−1^ and morphine 0.2 mg kg^−1^ was administered. The estimated total blood loss was 400 ml, which was replaced with 1100 ml of crystalloids.

**Fig. 3:**
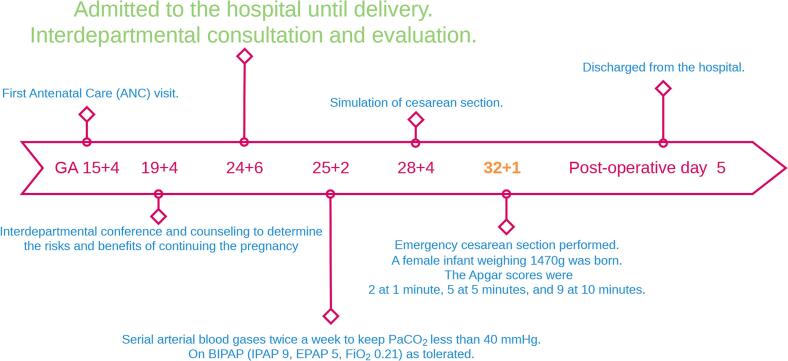
A timeline illustrating the sequence of events from patient admission to discharge.

Following the Caesarean delivery, the patient was transferred on ventilatory support to the intensive care unit. The patient's blood pressure was 110–140/70–80 mmHg, heart rate was 80–100 beats/min, respiratory rate was 18–24 breaths/min. The neuromuscular block was reversed with sugammadex 4 mg kg^−1^. The tracheal tube was removed, and there were no signs of respiratory insufficiency. Given the increased risk of fractures, breastfeeding was prohibited. The patient was discharged on postoperative day 4, and follow-up appointments were scheduled. A timeline illustrating the sequence of events from patient admission to discharge was shown in Fig. 3.

The child was diagnosed with respiratory distress syndrome and bilateral IVH grade 1. She was treated with CPAP for 3 days. High-flow nasal cannula (HFNC) was used for 10 days thereafter. After a total of 29 days in the hospital, the child was discharged home without complications.

## 3 Discussion

OI type XI or Bruck syndrome is a rare autosomal recessive disorder characterized by congenital contractures, early-onset fractures, short stature, limb deformities and progressive scoliosis [8]. This syndrome is caused by mutations in FKBP65 and PLOD2, which are involved in the crosslinking of telopeptides in type I collagen [3].

Among pregnant women with known OI type who reported having had a child, the majority (79 %) had type I OI [9]. The relatively small number of individuals with other phenotypes reporting pregnancies may indicate a lesser pregnancy rate among those more severely affected by the condition. Births to women with OI were associated with increased risks both for the mother and the fetus [10]. Several complications are reported, including respiratory insufficiency, difficult anesthesia management, antepartum hemorrhage, placenta abruption, intrauterine growth restriction and small-for-gestational-age infants, congenital malformation, and preterm birth and a higher likelihood of caesarean delivery [11,12].

Anesthetic management in pregnant women with OI is complex and requires careful consideration of several factors. Regional anesthesia is often preferred to avoid the potential complications associated with general anesthesia [9]. However, the published literature consists mostly of studies of patients with type I OI. The specific considerations regarding the vertebral malformations in our patient might have complicated the administration of neuraxial blocks [13]. Therefore, general anesthesia was selected to address the patient's specific condition to achieve a safe and effective delivery.

Previous studies have shown that individuals with OI may be prone to developing hyperthermia both during anesthesia and in the early postoperative period [[14], [15], [16], [17], [18]]. While the definitive cause of hyperthermia remains unclear, it is believed to differ from that of malignant hyperthermia [19,20]. Although the use of sevoflurane for maintenance of anesthesia can affect intraoperative temperature, recent literatures including a retrospective review of 252 orthopedic procedures, showed that inhalation anesthesia such as sevoflurane as well as TIVA could be used for the anesthesia for the patients with osteogenesis imperfecta. Furthermore, intraoperative hypothermia (<35.0 °C) was reported in 18 patients (8.4 %) in their study [21]. In this case, symptoms and signs compatible with malignant hyperthermia were not observed and intraoperative patient's temperature was normal. Further study is needed to develop appropriate anesthesia technique for OI patients who require surgery.

## 4 Conclusion

This case report presents the anesthetic management of a pregnant woman with OI type XI who underwent Caesarean delivery. Given the unique clinical challenges presented by this case, including the heightened risk of fractures, airway management complexities, and the potential for both maternal and fetal complications, our report offers landmark insights into the multidisciplinary collaboration and intraoperative strategies that led to develop safe positioning throughout the perioperative period and successful outcome.

## Contribution

KP, LS, ST, RK and SD drafted the manuscript. KP and LS clinically evaluated and managed the patient. All authors read and approved the final manuscript.

## Consent

Written informed consent was obtained from the patient for publication of this case report and accompanying images. A copy of the written consent is available for review by the Editor-in-Chief of this journal on request.

## Ethical approval

Ethical approval for this case report was granted by the Ramathibodi Human Research Ethics Committee (reference number COA.MURA2024/548) on August 9, 2024.

## Guarantor

Sasima Dusitkasem MD.

## Sources of funding

No funding was provided for the completion of this manuscript.

## Declaration of competing interest

All of the authors have no conflicts of interest to declare.
